# P046. ADHD and headache: observational study of case series

**DOI:** 10.1186/1129-2377-16-S1-A145

**Published:** 2015-09-28

**Authors:** Debora De Carlo, Guido de Rénoche, Massimo Ronchese, Luigi Bianchin, Barbara Bolzonella, Pier Antonio Battistella

**Affiliations:** Juvenile Headache Centre, Department of Woman and Child Health, University of Padua, Padua, Italy; Children and Adolescents Neuropsychiatry Unit ULSS 16, Padua, Italy

## Background

Attention deficit and hyperactivity disorder (ADHD) and headache are two very common diseases in childhood and both of them have an important impact on quality of life and academic performance [1]. In the literature there are many studies on psychopathology in headache, but the relationship between headache and ADHD is considered in few of them [2]. Recent studies have reported possible neural pathways and pathophysiological mechanisms that may underlie this relationship [3].

## Aim

Analysis of comorbility between ADHD and headache searching for the presence of ADHD trait in a population of headache patients.

## Subjects and methods

Observational study of case series based on collection of clinical-anamnestic data and on the administering of a standardized questionnaire (Strengths and Difficulties Questionnaire, SDQ) to evaluate the presence of ADHD traits in all the patients consecutively referred to the Juvenile Headache Centre of Padua (December 2014-May 2015). Inclusion criteria: age 5-18 years; diagnosis of primary headache, using the International Classification of Headache Disorders III, 2013 [4]: migraine without aura (MO) or with aura (MA), chronic migraine (CM), episodic (ETTH) or chronic tension-type headache (CTTH).

## Results

Total sample of 180 cases (81 M, 99 F) with mean age at interview of 11.8 years (8-18 years). Headache types: 120 migraine (M) (66.7%), 49 tension-type headache (TTH) (27.2%), 5 headaches with mixed pattern (M + TTH) (2.8%) and 6 other headaches (3.3%). M patients were divided into 107 MO (89.2%), 13 MA (10.8%); TTH were divided into 45 ETTH (91.8%) and 4 CTTH (8.2%). Family history for headache was present in 122/180 patients (71.8%), family history for M in 50/122 (41.0%). Prevalence of ADHD traits was 19.4% in SDQ questionnaires completed by parents and 21.3% in self-assessment SDQ questionnaires from children/adolescents. There was a low level of agreement between parents and children, reflecting heterogeneity symmetrical judgment between the two groups (p 0.53, K 0.53). There were no correlations with the diagnosis of headache or with other clinical features (sex, age of patients, age of onset, duration of illness, family history for headache); statistically significant relationships were found with the worsening of academic performance (p 0.001) and marginally with school absences (p 0.08).

## Conclusions

This study confirms most literature studies on the possible relationship between headache and ADHD, especially concerning the important impact on quality of life and academic performance [1–3]. It confirms the remarkable role of ADHD traits in the personal and family history of the juvenile patients affected by primary headaches.

Written informed consent to publish was obtained from the patient(s).
